# The status of medication literacy and associated factors of hypertensive patients in China: a cross-sectional study

**DOI:** 10.1007/s11739-019-02187-0

**Published:** 2019-10-24

**Authors:** Guiyue Ma, Aijing Luo, Zhiying Shen, Yinglong Duan, Shuangjiao Shi, Zhuqing Zhong

**Affiliations:** 1 Third Xiangya Hospital, Central South University, Changsha, 410013 China; 2 Xiangya School of Nursing, Central South University, Changsha, 410013 China; 3 Key Laboratory of Medical Information Research, Central South University, College of Hunan Province, Changsha, 410013 China

**Keywords:** Medication literacy, Hypertensive patients, Blood pressure control

## Abstract

The adverse consequence of low medication literacy is a major problem that threatens patients' health. The number of people with hypertension is increasing in China. We described the current situation of medication literacy of patients with hypertension in China and its related influencing factor. We conducted a cross-sectional study, which contains 590 hypertensive patients. Stratified sampling was adopted according to the hospital level in China. To determine the factors related to medication literacy, multiple linear regression analysis was used to determine associations between medication literacy of hypertensive patients and other factors. Among 590 respondents, results showed that they have poor medication literacy. Multiple linear regression analysis showed that level of education, annual income, occupation status, and type of medical insurance were significantly associated with medication literacy level of hypertensive patients. In addition, our study also demonstrates that we can identify the medication literacy level of hypertensive patients using the Chinese version Medication Literacy Scale for Hypertensive Patients. High medication literacy is an important factor for hypertensive patients to improve medication adherence, so as to better control blood pressure. We should pay attention to the improvement of medication literacy and take corresponding measures.

## Introduction

In China, hypertension has become an increasingly important topic, both for the public and the government [1]. Hypertension has been a great burden of disease. According to Report of cardiovascular diseases in China 2017 [2], there are currently 290 million people suffering from cardiovascular diseases, among whom 270 million are suffering from hypertension, accounting for 1/5 of the total number of people with hypertension in the world. Poor self-management and control of hypertension will lead to a variety of serious cardiovascular events, and its low awareness rate, low treatment rate, and low control rate greatly accelerate the occurrence and development of cardiovascular disease, causing great obstacles to the management and control of cardiovascular disease [3]. According to the report of the World Health Organization, 45% of the total deaths in China were caused by cardiovascular diseases in 2014 [4], and 50% of the cerebral apoplexy could be attributed to hypertension [5]. Besides, in the Summary of 2016 China cardiovascular disease report released in June 2017, the prevalence of people with hypertension over 18 years old is 25.2% in China [1]. It can be seen that Chinese hypertension patients tend to be younger, which further burdens the country and patients.

There are many treatments for high blood pressure, but top of the list is medication. The primary treatment for hypertension is still dominated by drug therapy, which plays an important role in the control of hypertension. Self-medication safety of patients with hypertension has always been the focus of researchers in the field of medicine and public health care. Because drug therapy is one of the most effective preventive and therapeutic measures for patients with hypertension [6]. Therefore, countries all over the world spend a lot of money on medicine every year [7, 8], especially in the treatment of hypertension [9]. In China, people spend more and more on medical care, which causes an increasing burden on people [10]. Studies show that 90% of hypertension patients can control their blood pressure at a safe level by taking drugs. However, the control rate of hypertension is still low, about 31% in developed countries and only 8.1% in China [11]. The reason is that patients' poor medication adherence leads to poor blood pressure control [12]. Nonadherence to medication is the major cause of poor blood pressure control, leading to cardiovascular disease and stroke [13, 14]. Even in patients with known complications such as myocardial infarction, poor medication adherence is also normal. Lau et al. [15] found in their study that less than half of hypertensive patients who took blood pressure lowering drugs for 1 year could still take the drugs consistently. Approximately 50% of CVD patients have poor adherence to their prescribed medications, which has adverse effects on morbidity, mortality, and recurrent cardiac complications, as well as higher costs [16, 17]. Many studies have shown that self-medication of antihypertensive drugs is poor in patients with hypertension, including poor adherence to antihypertensive drugs and medication errors [18]. Therefore, patients’ long-term regular use of antihypertensive drugs is an important way to stabilize blood pressure, control the disease and prevent serious complications. However, patients’ medication literacy is often low. As a result, the key to chronic disease control is the ability of patients to manage medications [19].

Medication literacy refers to the ability of an individual to self-medicate in a safe and appropriate way on the basis of obtaining, comprehending and communicating the information about the medications and correctly evaluating it, regardless of the manner in which the content is provided (e.g., written, verbal, and visual) [20]. The efficacy of drug therapy in patients with hypertension depends on their understanding of drug information and ability to obtain relevant support, which is conducive to patients' safe and rational self-medication. Therefore, high medication literacy is essential for successful treatment of patients with hypertension [21].

Currently, there is a lack of relevant medication literacy assessment tools for hypertensive patients in mainland China and there is also a lack of relevant cross-sectional studies on medication literacy of patients with hypertension, which is the gap we found. Therefore, on the basis of the pre-developed evaluation scale of medication literacy of hypertension patients, we designed Chinese version Medication Literacy Scale for Hypertensive Patients (C-MLSHP) to measure the medication literacy of hypertensive patients. In addition, the medication literacy of 540 cases of hypertensive patients in Changsha was investigated to analyze the relevant factors. This study aims to investigate the status of medication literacy in hypertensive patients in China and the influencing factors of medication literacy, so as to provide references for further improving the safe and reasonable self-medication of hypertensive patients.

## Methods

### Design and setting

This study was a cross-sectional survey conducted from August to December, 2016 with the purpose of understanding the factors related to medication literacy in patients with hypertension to improve the ability of safe and reasonable self-medication and blood pressure management. Based on the classification method of hospital level in China and the number of those hospitals of different levels, where patients with hypertension can be treated. Using the method of stratified sampling, 3 hospitals were selected from 15 tertiary hospitals and 2 from 11 secondary hospitals in Changsha City, Hunan Province. In addition, 3 community health service institutions were randomly selected from 15 communities in YueLu District of Changsha City, which sampling proportion is 20% individually.

### Study sample

Hypertensive patients who meet the inclusion criteria were participated to our study.

Inclusion criteria: (1) has been diagnosed as hypertension according to 2016 Revised Edition of Guideline to Hypertension Therapy in China; (2) over 18 years old; (3) time for antihypertensive medication over 2 weeks; (4) with clear consciousness and normal communication skills; and (5) agree to participate in this study and signed the informed consent. Exclusion criteria: (1) with severe associated disease of heart, brain, kidney and liver, and so on and (2) with mental illness.

### Measures

#### General information

Data collection was completed by four full-time graduate students who were trained before data collection to ensure the quality and scientific nature of the data. The members of the study group, after obtaining the permission of the relevant officers of the various investigation centers, selected the subjects that met the inclusion and exclusion criteria; the study population were informed the purpose and significance of the study and signed the informed consent. Participants were asked to complete a self-made general demographic questionnaire regarding their age, gender, education, income by year, marital status, occupation, location, type of medical insurance, history of hypertension, family history of hypertension, complication of hypertension, the number of antihypertensive drugs being taken, and the number of people who live together. The questionnaires were required to be filled out and collected on the spot, and the completion of the questionnaire was been checked, reason was been asked if there was an omitted item in the questionnaire, then the omission had to been filled as complete as possible by participants.

### Chinese version Medication Literacy Scale for Hypertensive Patients (C-MLSHP)

We designed the Chinese version Medication Literacy Scale for Hypertensive Patients (C-MLSHP) to measure the medication literacy of hypertensive patients, which was based on four dimensions: medication knowledge literacy, skill literacy, attitude literacy, and practice literacy, a total of 37 items. The contents include nine items for medication knowledge literacy, including three factors of self-medication knowledge, knowledge of hypertension, knowledge of hypertension therapy; eight items for attitude literacy, including two factors of attitude to self-medication and disease of hypertension; seven items for skill literacy, including two factors of reading, comprehension skill and calculating skill; 13 items for practice literacy, including four factors of compliance behavior, behavior of medication decision, behavior of blood pressure monitoring, medication information-seeking, and disseminating behavior. The Cronbach’s α coefficient of the scale is 0.849, and the Cronbach’s *α* coefficient of each subscale is between 0.744 and 0.783. The split-half reliability of the whole scale is 0.893 and the split-half reliability of subscales are from 0.793 to 0.872; The test–retest reliability of the whole scale is 0.968 and the test–retest reliability of subscales are from 0.880 to 0.959; I-CVI was 0.833–1.000, S-CVI was 0.968.

The total scores range from 0 to 37, of which the correct answer of each dimension of knowledge and skill was 1, and the wrong answer was 0. In the dimension of attitude and practice, the Likert-5 scores were 1.0, 0.75, 0.5, 0.25, 0. Among them, there are five reverse items in attitude dimension and one reverse item in practice dimension, and the score is opposite. In general, the higher the score, the higher the medication literacy.

### Data analysis

All the data in this study were recorded by two persons. Excle2016 was used to record and store the data. We examined the collected data, then collate and encode it. Means and standard deviations were used to describe quantitative variables, frequencies and percentages were used to describe categorical variables. *T* test, LSD *t* test, and multiple linear regression analysis were used for inferential statistics. Statistical analysis was performed using SPSS version 22. The criterion of significance is *α* = 0.05, which makes sense if *P* < 0.05.

## Results

### General information

Totally, 590 hypertensive patients were surveyed in this study. 590 questionnaires were sent out and 590 were collected and response rate was 100%. Nevertheless, there were 540 validated questionnaires after checking, so the validated rate was 91.7%. Approximately 11.5% of participants had completed junior college, 18.7% of participants earned more than 100,000 a year, 75.6% of participants occupied, 41.9% of participants have medical insurance for urban workers. Other general information about participants are shown in Table 1.

**Table 1:** Demographics of hypertensive patients (*n* = 540)

	Items	*n*	%
Age	18–44	58	10.70
	45–59	172	31.90
	≥ 60	310	57.40
Gender	Male	298	55.20
	Female	242	44.80
Education level	Primary or below	149	27.60
	Junior middle school	145	26.90
	High school or polytechnic school	101	18.70
	Junior college	62	11.50
	Bachelor degree or above	83	15.40
Annual Income	< 10,000/year	94	17.40
	10,000–29,000/year	106	19.63
	30,000–49,000/year	146	27.04
	50,000–99,000/year	93	17.20
	≥ 100,000/year	101	18.70
Marital status	Married	479	88.70
	Unmarried	10	1.90
	Divorced or widowed	51	9.50
Occupation	Occupied	374	75.60
	Retired	128	23.70
	Jobless	38	7.00
Habitation	Urban	310	57.40
	Country	230	42.60
Type of medical insurance	New rural cooperative medical insurance	206	38.10
	Medical insurance for urban workers	226	41.90
	Medical insurance for urban residents	86	15.90
	Other	15	2.80
	Without medical insurance	7	1.30
A history of hypertension	< 3 years	101	18.70
	3–4 years	90	16.70
	5–9 years	144	26.70
	≥ 10 years	205	38.00
Family history of hypertension	Yes	348	64.40
	No	192	35.60
Hypertension complication	Yes	146	27.00
	No	394	73.00
	One or none	333	61.70
The number of antihypertensive medications being taken	2–3 kinds	185	34.30
	4 kinds or above	22	4.10
	One or none	24	4.40
The number of people who live together	2–4	346	64.10
	5–7	142	26.30
	8 or above	28	5.20

### Scores of each dimension of medication literacy in hypertensive patients

The medication literacy of hypertensive patients has four dimensions, and the results show that hypertensive patients have the highest score in knowledge literacy and the lowest score in skill literacy, as shown in Table 2.

**Table 2:** Scores in each dimension of medication literacy of hypertensive patients

Dimensions	Range of score	Overall average score	Average score of items	sequence
Knowledge literacy	0.00–9.00	6.28 ± 2.21	0.70 ± 0.25	1
Attitude literacy	2.00–8.00	5.36 ± 1.20	0.67 ± 0.15	2
Skill literacy	0.00–7.00	4.51 ± 2.15	0.64 ± 0.31	4
Practice literacy	1.00–12.75	8.46 ± 1.95	0.65 ± 0.15	3
Medication literacy	11.50–35.5	24.61 ± 5.13	0.67 ± 0.14	

### Analysis on influencing factors of medication literacy of hypertensive patients

#### Scores on medication literacy assessment scale of hypertensive patients with different characteristics

The score and characteristics of each dimension of the medication literacy assessment scale of hypertensive patients are different, and the results of t test and LSD *t* test in each item are shown in Table 3.

**Table 3:** Scores on medication literacy assessment scale of hypertensive patients of different characteristics

Factors	Items	Knowledge literacy	Attitude literacy	Skill literacy	Practice literacy	Scores on medication literacy
Age	18–44	7.16 ± 1.63	5.17 ± 1.35	5.53 ± 1.55	9.25 ± 1.70	27.10 ± 4.77
	45–59	6.40 ± 2.17^a^	5.40 ± 1.24	4.73 ± 2.10^a^	8.33 ± 1.93^a^	24.87 ± 5.02^a^
	≥ 60	6.05 ± 2.29^a^	5.37 ± 1.15	4.19 ± 2.20^a^	8.38 ± 1.97^a^	24.01 ± 5.11^a^
*F* value		6.539	0.878	11.300	5.430	9.510
*P* value		0.002	0.416	0.000	0.000	0.000
Gender	Male	6.66 ± 2.06	5.35 ± 1.22	4.77 ± 2.02	8.57 ± 1.99	25.35 ± 4.92
	Female	5.81 ± 2.30	5.38 ± 1.18	4.19 ± 2.26	8.33 ± 1.88	23.71 ± 5.25
*T* value		4.524	0.288	3.137	1.424	3.737
*P* value		0.000	0.773	0.002	0.155	0.000
Degree of education	Primary or below	5.21 ± 2.20	5.15 ± 1.17	3.95 ± 2.30	8.24 ± 1.99	22.55 ± 4.99
	Junior middle school	5.92 ± 2.20^a^	5.44 ± 1.21	4.14 ± 2.30	8.14 ± 1.88	23.64 ± 5.28
	Senior school or technical secondary school	6.49 ± 1.97^a^	5.36 ± 1.14	4.69 ± 2.07^a^	8.73 ± 1.66	25.27 ± 4.19^ab^
	Junior college	7.31 ± 1.66^abc^	5.44 ± 1.26	5.10 ± 1.64^ab^	8.86 ± 2.08	26.70 ± 4.49^abc^
	College degree or above	7.84 ± 1.59^abc^	5.55 ± 1.23	5.48 ± 1.47^abc^	8.79 ± 2.11	27.67 ± 4.43^abc^
*F* value		28.482	1.943	9.733	3.270	20.234
*P* value		0.000	0.102	0.000	0.012	0.000
Annual income	≤ 10,000/year	5.45 ± 2.53	5.19 ± 1.14	4.11 ± 2.22	7.94 ± 1.92	22.68 ± 4.79
	10,000–29,000/ year	5.55 ± 1.09	5.33 ± 1.09	4.03 ± 2.36	8.13 ± 2.05	23.04 ± 5.39
	30,000–49,000/ year	6.57 ± 2.01^ab^	5.58 ± 1.20	4.54 ± 2.20	8.70 ± 1.77^a^	25.40 ± 5.06^ab^
	50,000–99,000/year	6.80 ± 1.98^ab^	5.21 ± 1.35	5.18 ± 1.85^a^	8.64 ± 2.22^a^	25.83 ± 5.09^ab^
	≥ 100,000/year	6.95 ± 1.76^ab^	5.38 ± 1.19	4.72 ± 1.84	8.77 ± 8.46^a^	25.82 ± 4.36^ab^
*F* value		11.249	2.154	4.832	3.908	10.017
*P* value		0.000	0.073	0.001	0.004	0.000
Marital status	Unmarried	6.40 ± 2.12	5.05 ± 1.09	5.60 ± 1.58	9.28 ± 1.71	26.33 ± 4.84
	Married	6.33 ± 2.18	5.34 ± 1.22	4.53 ± 2.14	8.44 ± 1.98	24.65 ± 5.16
	Divorced or widowed	5.78 ± 2.46	5.60 ± 1.05	4.08 ± 2.25	8.51 ± 1.65	23.98 ± 4.91
*F* value		1.441	1.431	2.355	0.926	0.962
*P* value		0.238	0.240	0.096	0.397	0.383
Occupational status	On-the-job	6.31 ± 2.24	5.38 ± 1.22	4.62 ± 2.17	8.52 ± 1.91	24.83 ± 5.16
	Retired	6.63 ± 2.05	5.48 ± 1.09	4.41 ± 2.08	8.46 ± 2.10	24.97 ± 4.90
	Jobless	4.89 ± 1.93^ab^	4.78 ± 1.25^ab^	3.76 ± 2.03^a^	7.86 ± 1.64	21.29 ± 4.51^ab^
*F* value		9.322	5.236	2.915	2.032	8.883
*P* value		0.000	0.006	0.055	0.132	0.000
Residence	Urban	6.68 ± 2.03	5.46 ± 1.20	4.68 ± 2.01	8.54 ± 1.96	25.37 ± 4.78
	Rural areas	5.75 ± 2.33	5.22 ± 1.19	4.27 ± 2.31	8.35 ± 1.92	23.60 ± 5.41
*T* value		4.910	2.323	2.201	1.140	4.022
*P* value		0.000	0.021	0.028	0.255	0.000
Medical insurance type	Without medical insurance	3.29 ± 1.70	4.25 ± 0.85	1.86 ± 2.34	7.39 ± 2.31	16.79 ± 4.90
	New rural cooperative medical insurance	5.62 ± 2.33^a^	5.29 ± 1.25^a^	4.43 ± 2.33^a^	8.28 ± 1.92	23.63 ± 5.46^a^
	Medical insurance for urban workers	7.13 ± 1.85^a^	5.45 ± 1.20^a^	4.92 ± 1.82^a^	8.55 ± 2.08	26.05 ± 4.59^a^
	Medical insurance for urban residents	5.81 ± 1.94^a^	5.31 ± 1.10^a^	3.87 ± 2.22^a^	8.70 ± 1.62	23.70 ± 4.56^a^
	Others	6.73 ± 2.34^a^	5.75 ± 0.86^a^	4.33 ± 1.88^a^	8.65 ± 1.71	25.47 ± 4.00^a^
*F* value		19.779	2.423	6.972	1.448	12.119
*P* value		0.000	0.047	0.000	0.217	0.000
History of hypertension	< 3 year	6.07 ± 2.24	5.20 ± 1.25	4.61 ± 2.24	8.67 ± 1.81	24.55 ± 5.30
	3–4 year	5.84 ± 2.11	5.23 ± 1.21	4.84 ± 2.14	8.38 ± 1.87	24.29 ± 5.07
	5–9 year	6.47 ± 2.16	5.34 ± 1.22	4.37 ± 2.27	8.40 ± 2.04	24.57 ± 5.23
	≥ 10 year	6.45 ± 2.26	5.52 ± 1.14	4.41 ± 2.01	8.44 ± 1.98	24.82 ± 5.02
*F* value		2.243	2.291	1.165	0.497	0.24
*P* value		0.082	0.077	0.322	0.684	0.868
Family history of hypertension	Yes	6.57 ± 2.19	5.36 ± 1.24	4.67 ± 2.09	8.44 ± 2.04	25.04 ± 5.12
	No	5.76 ± 2.16	5.36 ± 1.14	4.22 ± 2.23	8.50 ± 1.77	23.84 ± 5.07
*T* value		4.142	0.043	2.344	0.345	2.611
*P* value		0.000	0.966	0.019	0.730	0.009
Hypertension complication	Yes	6.10 ± 2.39	5.35 ± 1.11	4.27 ± 2.32	8.35 ± 2.20	24.07 ± 5.68
	No	6.35 ± 2.14	5.37 ± 1.23	4.60 ± 2.08	8.50 ± 1.84	24.82 ± 4.90
*T* value		1.156	0.144	1.597	0.827	1.516
*P* value		0.248	0.885	0.111	0.409	0.130
The number of antihypertensive medication being taken						
	1 kind	6.43 ± 2.10	5.29 ± 1.18	4.63 ± 2.13	8.45 ± 1.95	24.80 ± 4.85
	2–3 kinds	6.17 ± 2.31	5.47 ± 1.22	4.31 ± 2.20	8.54 ± 1.93	24.49 ± 5.57
	4 kinds or above	5.05 ± 2.59^a^	5.53 ± 1.22	4.32 ± 1.94	7.95 ± 1.96	22.85 ± 5.15
*F* value		4.485	1.616	1.389	0.883	1.578
*P* value		0.012	0.200	0.250	0.414	0.207
The number of people who live together	1 or below	6.33 ± 2.41	5.14 ± 1.12	3.79 ± 2.25	8.75 ± 1.63	24.01 ± 5.50
	2–4	6.44 ± 2.21	5.43 ± 1.22	4.61 ± 2.13	8.59 ± 1.92	25.07 ± 5.16
	5–7	6.02 ± 2.07	5.24 ± 1.14	4.43 ± 2.21	8.27 ± 2.05	23.96 ± 4.96
	8 or above	5.61 ± 2.54	5.34 ± 1.27	4.25 ± 1.96	7.61 ± 1.72^ab^	22.80 ± 4.58
*F* value		2.151	1.166	1.364	2.916	2.987
*P* value		0.093	0.322	0.253	0.034	0.031

^a^Compared with the first layer, *P* < 0.05

^b^Compared with the second layer, *P* < 0.05

^c^Compared with the third layer, *P* < 0.05

#### Multiple linear regression analysis on influencing factors of medication literacy of hypertensive patients

In this study, the independent variables are age, gender, education level, annual income, marital status, occupation, habitation, type of medical insurance, a history of hypertension, family history of hypertension, hypertension complication, the number of antihypertensive medications being taken, the number of people who live together. The dependent variables are score of medication literacy of hypertensive patients and score of each dimension of medication literacy (specific assignment for variables are in Table 4). Multiple linear regression analysis was used. The results show that the education level, annual income, occupation, medical insurance type are the influencing factors on medication literacy of hypertensive patients, which are shown on Table 5.

**Table 4:** Variables assignment of analysis on influencing factors of medication literacy of hypertensive patients

Variables	Assignment
Gender	Male = 1; female = 2
Age group	18–44 years old = 1; 45–60 years old = 2; ≥ 60 years old = 3
Marital status	Referring to “unmarried” as dumb variable: M1 = married (1, 0), M2 = divorced or widowed (0, 1)
Degree of education	Primary level and below = 1; Junior middle school = 2; Senior school or technical secondary school = 3; Junior College = 4; College degree or above = 5
Occupational status	Referring to the retired as dumb variable: P1 = on-the-job (1, 0), P2 = jobless (0, 1)
Annual income	≤ 10,000/year = 1; 10,000–29,000/year = 2; 30,000–49,000/year = 3; 50,000–99,000/year = 4; ≥ 100,000/year = 5
Residence	Urban area = 1; Rural area = 2
The number of people who lived together	1 or below = 1; 2–4 persons = 2; 5–7 persons = 3; 8 or above = 4
Medical insurance type	Referring to “without medical insurance” as dumb variable: I1 = Medical insurance for urban workers (1, 0, 0, 0), I2 = Medical insurance for urban residents (0, 1, 0, 0), I3 = New rural cooperative medical insurance (0, 0, 1, 0), I4 = others (0, 0, 0, 1)
History of hypertension	< 3 years = 1; 3–4 years = 2; 5–9 years = 3; ≥ 10 years = 4
Family history of hypertension	Yes = 1; no = 2
Hypertension complication	Yes = 1; no = 2
Kinds of antihypertensive medication being taken	1 or below = 1; 2–3 kinds = 2; 4 or above = 3

**Table 5:** Results of multiple linear regression analysis on each dimension of medication literacy of hypertensive patients

Model summary				
Model	R	R2	Adjusted R2	Error of standard estimate
1	0.349^a^	0.122	0.117	5.278

	B	SE	Beta	*T*	*P*
(constant)	15.194	2.420		6.279	0.000
Degree of education	0.941	0.188	0.256	5.006	0.000
Annual income	0.488	0.170	0.128	2.868	0.004
P1 on-the-job	0.115	0.504	0.010	0.229	0.819
P2 jobless	− 2.429	0.911	− 0.121	− 2.667	0.008
I1 Medical insurance for urban workers	7.562	1.814	0.728	4.169	0.000
I2 Medical insurance for urban residents	6.557	1.848	0.468	3.549	0.000
I3 New rural cooperative medical insurance	7.212	1.806	0.684	3.993	0.000
I4 others	7.581	2.155	0.243	3.518	0.000

^a^Predictive variables: (constant), education level, occupational status, and medical insurance

## Discussion

### The level of medication literacy of patients with hypertension needs to be improved

There are no studies that investigate the influencing factors related to medication literacy of hypertension. Furthermore, failure to adhere to medication is a preventable cause of treatment failure [6]. The goal of our study is to describe the current situation of medication literacy of patients with hypertension in China and its related influencing factor. The results showed that they scored totally (24.61 ± 5.13) in medication literacy, of which each dimension was scored an average of (0.67 ± 0.14). Results show that the medication literacy of patients with hypertension is not high. Therefore, it is particularly necessary to explore the influencing factors of medication literacy in hypertensive patients.

Furthermore, from the scoring results of each dimension of medication literacy, we can see that the average score of knowledge literacy is the highest, followed by the scores of attitude, practice and skill literacy. It is concluded that patients with hypertension have a better understanding of the disease of hypertension and knowledge of antihypertensive drugs than skills, attitudes, and practices of self-medication. Moreover, the poor quality of self-medication practice in hypertensive patients indicates that they cannot put their medication knowledge into safe and reasonable self-medication practice. The study also proved that we can measure the medication literacy level of hypertensive patients using the Chinese version Medication Literacy Scale for Hypertensive Patients (C-MLSHP).

### Influencing factors of level of medication literacy of hypertensive patients

#### Education level influencing on level of medication literacy of hypertensive patients

The results showed that the differences of medication literacy in hypertensive patients with different levels of education were mainly manifested in the two dimensions of knowledge and skill literacy, which was similar to Newman et al. [22]. However, Lin et al. [23] found that knowledge related to disease was irrelevant to medication adherence. In the study, the knowledge and skill literacy of patients with education of high school or technical secondary school was significantly higher than that of patients with junior middle school or below. Education can increase patient knowledge, which is inconsistent with another study [24]. High blood pressure skills can also be improved through education [25]. Hanafi et al.’s [26] studies have shown that health education not only can improve knowledge, but also can improve attitude and practice. Moreover, Toselli et al. [27] found that for North African women who moved to Italy, socioeconomic variables had a significant impact on obesity, with low education associated with an increased risk of obesity. It also suggests that low literacy can affect people's behavior. Results of multiple linear regression indicate that the educational level of patients can affect their level of medication literacy. With the gradual increasing of education level, the level of medication literacy of patients with hypertension has also increased, which were similar to the research in medication literacy of other populations [28]. The higher the level of education, the stronger and better the understanding of language and words of medical staff. However, this study indicates that the educational level is relatively low, especially for people with high blood pressure in junior high school or below. Effective measures should be taken to improve the medication literacy of the target population, paying particular attention to their knowledge and skills.

#### Influence of level of income by year on medication literacy of patients with hypertension

Different levels of annual income are also influencing factors affecting the level of medication literacy in hypertensive patients. With the increase of annual income, the level of medication literacy of the patients will be improved. It was different in dimensions of knowledge, skills and practical behavior literacy between those with annual income of less than 30,000 yuan and of more than 30,000 yuan (*P* < 0.05), which suggested that hypertensive patients with higher annual incomes were more likely to pay more attention to promoting health level and they value the health education of medical staff than those with lower annual incomes. Socioeconomic factors have been shown to affect health through matter [20]. Maybe people with high salaries are willing to spend a lot of money on health care and health promotion. In this study, 200 patients with an annual income of less than 30,000 yuan, among which 76% have a junior middle school education or below, indicating that patients with a low annual income generally have a low educational level. Nonetheless, hypertension patients with lower education and better cognitive status often benefit the most from intervention [30]. Therefore, hypertensive patients with lower annual income are also the focus group for improving medication literacy. However, differences in annual income did not significantly affect hypertension patients' attitudes to disease and medication management. Patients' attitudes may be related to symptoms of high blood pressure, their level of attention, and their use of medications, just like a Belgian study [31]. The influence of economic income on the attitude dimension of medication literacy may not be significant.

#### Influence of occupational status on medication literacy of patients with hypertension

The results showed that the knowledge level of unemployed patients was lower than that of retired and occupied patients, including the knowledge of disease and medication, attitudes towards disease and medication administration, and skills for medication administration. Results of multiple linear regression also suggest that occupational status is one of the influencing factors of medication literacy of hypertensive patients. Among all the unemployed residents, 92.1% have a junior high school education or below, and 60.5% have an education above the age of sixty, which indicates that attention should be paid to the improvement of medical literacy of the unemployed. There is also a study abroad that shows that people who are employed have a higher level of education [32]. In addition, Eikemo et al. [33] found that among Greek-born immigrant groups, women are more likely to develop non-communicable diseases, which may be due to occupational factors. Thus, the importance of occupational factors can be seen.

#### Influence of type of medical insurance on medication literacy of patients with hypertension

Among the hypertensive patients with different medical insurance types, the knowledge level of hypertensive patients with urban workers' medical insurance was the highest, followed by the new rural cooperative medical insurance and urban residents' medical insurance, and the knowledge level of hypertensive patients without medical insurance was the lowest. The results of multiple linear regression analysis also show that the type of medical insurance is one of the factors affecting the knowledge level of hypertension patients, which may be because patients with urban residents' medical insurance have better economic conditions [34–36]. Furthermore, due to better medical reimbursement in such two kinds of medical insurance, patients may take less concern of medical expenses and focus mainly on their disease. Therefore, the medication literacy of hypertensive patients without medical insurance should be better promoted.

### Recommendations for improving the level of medication literacy in hypertensive patients

Carry out comprehensive screening of medication literacy for hypertensive patients, and determine the key intervention population. First of all, it is necessary to use medication literacy assessment tools for screening and evaluating medication literacy of hypertensive patients to identify the urgency and necessity of medication literacy promotion strategies. At the same time, specific measures should be taken to identify individual differences to better improve the safety of drug use. In addition, attention should be paid to the low education, low income, unemployment, no insurance, to improve their medication literacy. Health education is mainly to improve the quality of medication literacy in patients with hypertension through effective intervention measures by health service personnel, which is very important. Therefore, it is necessary to carry out systematic and professional training for health service personnel, so as to make health education more comprehensive and conducive to patients' acceptance. Meanwhile, we need government oversight and policies to help primary care providers provide sustainable self-medication guidance to patients.

## Limitations

Our study has some limitations. Self-report bias exists in this study. In addition, selection bias may exist in patients with hypertension as most of them are elderly. In addition, the analysis of influencing factors related to medication literacy may be incomplete, and cross-sectional study designs may not be able to determine the causal relationship between the variables of interest. The results may not apply to other populations.

## Conclusion

Adequate medication literacy is helpful for the control of blood pressure, but medication literacy of hypertensive patients is generally low. Therefore, medication literacy of hypertensive patients needs to be improved. Attention should be paid to the low education, low income, unemployment, no insurance, to improve their medication literacy. Health education is an important measure to ensure the safe self-medication of hypertensive patients. Besides, screening patients in need of help for intervention, systematic training of health service personnel and comprehensive mobilization of medical resources can provide a way to improve the level of medication literacy of hypertensive patients.
